# Underutilized Agricultural Co-Product as a Sustainable Biofiller for Polyamide 6,6: Effect of Carbonization Temperature

**DOI:** 10.3390/molecules25061455

**Published:** 2020-03-24

**Authors:** Thomas Balint, Boon Peng Chang, Amar K. Mohanty, Manjusri Misra

**Affiliations:** 1 Bioproducts Discovery and Development Centre, Department of Plant Agriculture, Crop Science Building, University of Guelph, 50 Stone Road East, Guelph, ON N1G 2W1, Canada; tbalint@uoguelph.ca (T.B.); changb@uoguelph.ca (B.P.C.); 2 School of Engineering, Thornbrough Building, University of Guelph, 50 Stone Road East, Guelph, ON N1G 2W1, Canada

**Keywords:** nylon 6,6, pyrolysis, biobased composite, carbonaceous natural fiber, low-cost biofiller, industry co-product

## Abstract

Polyamide 6,6 (PA66)-based biocomposites with low-cost carbonaceous natural fibers (i.e., soy hulls, co-product from soybean industry) were prepared through twin-screw extrusion and injection molding. The soy hull natural fiber was pyrolyzed at two different temperatures (500 °C and 900 °C denoted as BioC500 and BioC900 respectively) to obtain different types of biocarbons. The BioC500 preserved a higher number of functional groups as compared to BioC900. Higher graphitic carbon content was observed on the BioC900 than BioC500 as evident in Raman spectroscopy. Both biocarbons interact with the PA66 backbone through hydrogen bonding in different ways. BioC900 has a greater interaction with N-H stretching, while BioC500 interacts strongly with the amide I (C=O stretching) linkage. The BioC500 interrupts the crystallite growth of PA66 due to strong bond connection while the BioC900 promotes heterogeneous crystallization. Dynamic mechanical analysis shows that both biocarbons result in an increasing storage modulus and glass transition temperature with increasing content in the BioC/PA66 biocomposites over PA66. Rheological analysis shows that the incorporation of BioC900 results in decreasing melt viscosity of PA66, while the incorporation of BioC500 results in increasing the melt viscosity of PA66 due to greater filler–matrix adhesion. This study shows that pyrolyzed soy hull natural fiber can be processed effectively with a high temperature (>270 °C) engineering plastic for biocomposites fabrication with no degradation issues.

## 1. Introduction

Polyamide 6,6 (PA66) (also known as nylon 6,6) is an engineering thermoplastic that is usually employed in engineering applications and durable auto parts due to its excellent properties such as low density, good abrasion resistance, low friction, exceptional thermal stability, and dimensional stability [1]. Despite its high performance and high strength-to-weight ratio, PA66 is high in cost compared to PA6 [2] and other commercial engineering plastics (e.g., 26% higher than nylon 6, and 47% higher than engineering polyester, polybutylene terephthalate (PBT) [3]. Therefore, low cost-fillers could potentially offset the high cost of PA66.

Polymer composites allow for convenient tailorability and flexible design of properties. There has been much interest on using high-performance carbon-based materials as filler for polymer composite fabrication, such as graphene [4] and carbon nanotubes [5]. Although they promise excellent reinforcing performance, their complex extraction and purification processes, as well as low yield makes them not cost-effective or feasible for composite application. In recent years, research using low-cost agriculture co-products, food wastes, as well as other environmentally friendly fillers for composite applications has expanded rapidly due to global awareness and promotion of sustainable development. 

The conversion of underutilized agricultural residue biomasses into products with high-value applications has been a growing area of research in recent years [6,7]. Some of the potential applications include water treatment and purification [8,9,10], nanoparticle production [11], hydrogels [12], and high-performance nanocellulose [13,14]. Soybeans are cultivated worldwide to be used as feed for livestock, and as a source of protein in the human diet, along with other applications [15]. In 2016 the global average annual soybean production was over 313 million tons [16], with approximately 10 percent of this value being hulls [17]. During processing, soy hulls (by-product) from soybean processing are removed from soybeans and commonly added as filler to animal feed, used in other applications, or sent to disposal or landfill [17,18]. Soy hulls are typically composed of 29–51% cellulose, 1–4% lignin, and 10–20% hemicelluloses with the remainder primarily consisting of proteins and pectins [19,20]. A proper utilization of this waste as a biobased filler (biofiller) for composite applications could reduce the environmental strain. A number of studies have been dedicated to utilizing soy hull waste for polymer composite applications with promising results [21,22,23,24]. 

Biosourced carbons (biocarbon) are low-cost carbon-based materials derived from different biomasses which are gaining popularity due to their carbon sequestration to the benefit of the environment [25,26], which combats global climate change. Natural fibers or raw natural biomass are not suitable as reinforcement for high processing temperature engineering plastics such as nylon 6, nylon 6,6, PBT and polyethylene terephthalate (PET) due to degradation. However, this can be overcome by properly controlling the charring or carbonization of natural fiber with pyrolysis techniques. The carbonization process turns natural fiber into a more thermally stable material. The main components of natural fiber mentioned above (i.e., cellulose, hemicellulose, waxes and lignin) degrade and burn off during pyrolysis, leaving a thermally stable aromatic carbon structure. This carbonized natural fiber is able to withstand the high processing temperatures of engineering plastics better as compared to its raw form. Vold et al. compared the properties of PA6-based biocomposites filled with carbonized agricultural wastes and their raw forms [27]. The carbonized biofillers showed significant improvement on mechanical strength as compared to non-carbonized biofiller-reinforced PA6 biocomposites due to the superior thermal stability of the carbonized biofillers when processed at high temperature. Zhang et al. found that carbonized rice husk-reinforced high-density polyethylene (HDPE) exhibited better mechanical performance than powdered rice husk-reinforced HDPE due to better affinity with non-polar HDPE after pyrolysis [28].

This pyrolyzed material’s properties can be further tailored with various technological processes for different applications (i.e., highly graphitic engineered biocarbon, and activated carbon). Syngas and bio-oil are the two main by-products from the pyrolysis process which can be also exploited for appropriate applications for a greener environment. It has been observed that the incorporation of biocarbon may positively influence some properties of different polymers [29,30,31,32,33,34,35]. Biocarbon-based biocomposites possess a high potential to be used in nylon-based automotive parts [36,37]. Despite several studies on the effects of biocarbon on the performance of various polymers, the molecular interactions between natural fibers carbonized at different temperatures and engineering polymers with high processing temperatures, particularly PA66, are not widely reported. This work aims to examine the effect of different pyrolysis conditions on the thermal and crystallization behavior, dynamic mechanical properties, rheological properties, as well as molecular interactions of soy hull biocarbon/PA66 biocomposites in order to assess the potential economic viability of this low-cost filler for PA66 for value-added applications in the future. 

## 2. Results and Discussion 

### 2.1. Biocarbon Morphology and Characterization 

**Figure 1:**
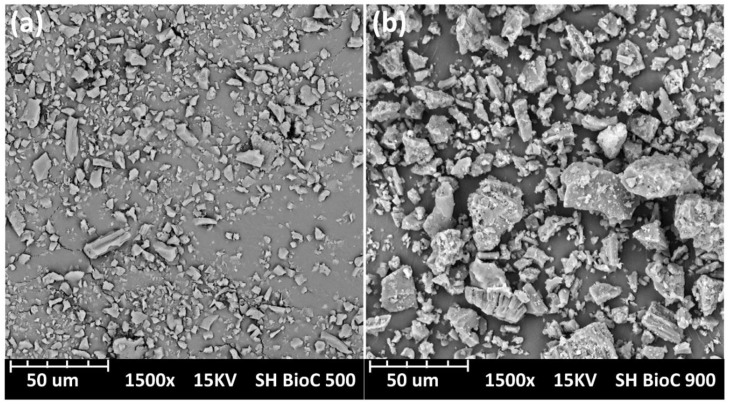
Particle size comparison at different pyrolysis temperatures of soy hull biomass (After ball milling and sieving) (**a**) 500 °C BioC and (**b**) 900 °C BioC.

The morphologies of soy hull biomass after being pyrolyzed with different conditions and ball milled are depicted in Figure 1. It was observed that the low-temperature biocarbon pyrolyzed at 500 °C (BioC500) displayed a smaller particle size than the high-temperature biocarbon pyrolyzed at 900 °C (BioC900) under the same magnification. Both biocarbons showed an accumulation of a cluster of particles. It can be seen that the average size of the low pyrolysis temperature biocarbon, BioC500 is approximately 20 µm while the size of the high pyrolysis temperature biocarbon, BioC900 particles is around 50 µm. This phenomenon can be interpreted as the formation of highly ordered carbon packing structures (graphitization) at high carbonization temperatures which yields carbon with higher hardness and denser structure. The particle size of BioC900 remained bigger as compared to BioC500 after ball milling for the same duration and conditions due to its higher stiffness and graphitic structure. 

**Figure 2:**
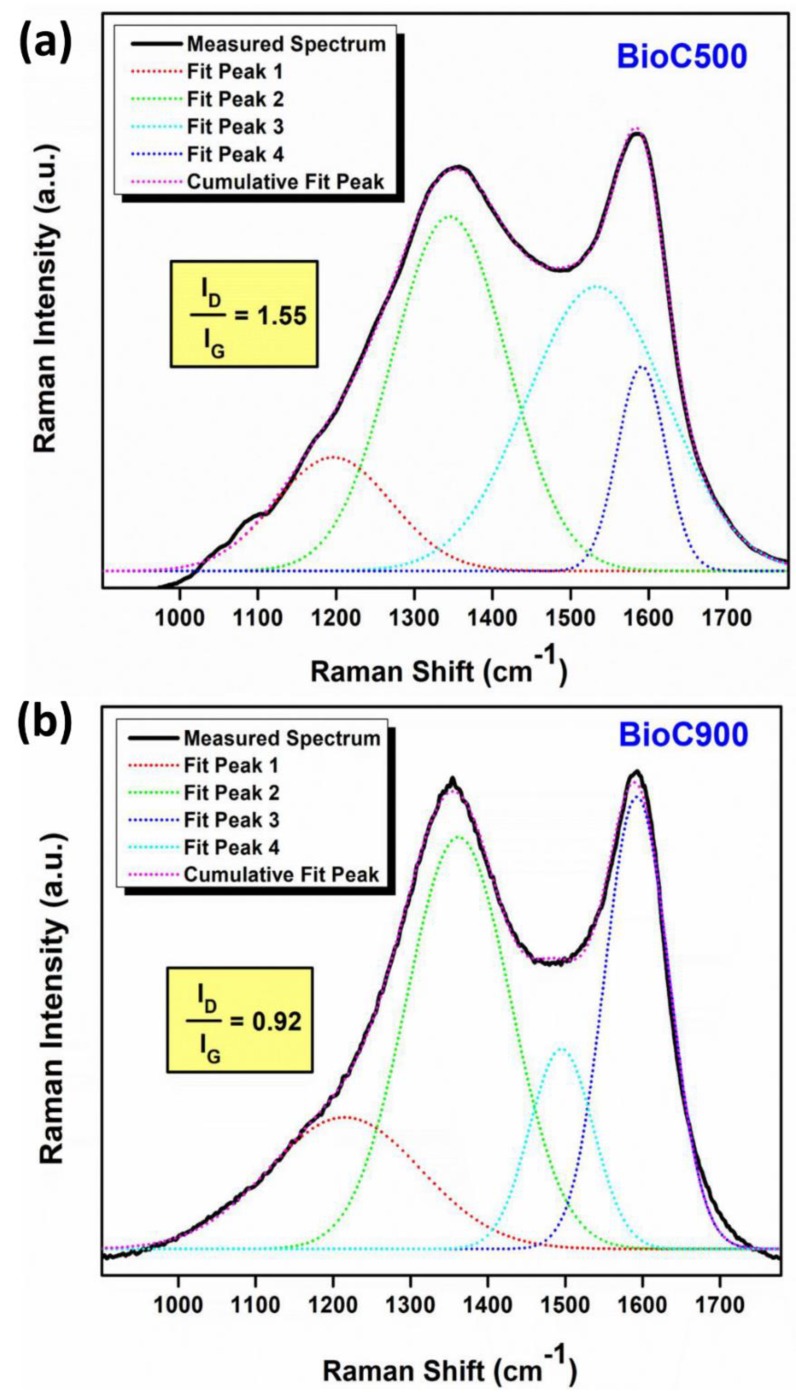
Raman spectroscopy of (**a**) 500 °C and (**b**) 900 °C soy hull biocarbon.

The degree of carbonization of both carbonized soy hulls were further investigated with Raman spectroscopy. The D-band (disordered carbon) and G-band (highly ordered carbon or graphitic carbon) of the carbon materials can be detected through the Raman scattering. Figure 2 illustrates the Raman spectra of the biocarbon at different pyrolysis temperatures. It can be seen that G-band intensity is higher than that of the D-band intensity for high-temperature biocarbon, while the reverse case was observed for the low-temperature biocarbon. The ratio of I_D_/I_G_ of BioC500 is 1.55, while for BioC900 is 0.92. This indicates the high pyrolysis temperature biocarbon contains a higher amount of orderly carbon structure as compared to the low-temperature biocarbon. The organic carbon content increases with increasing pyrolysis temperature, while the oxygen and hydrogen content decreased [38]. Therefore, the H/C ratio, as well as the degree of carbonization increases with increasing processing temperature [39,40]. 

Furthermore, the highly-dense carbon structure of high-temperature biocarbon was also reflected in the density of the developed biocomposites. The specific gravity of the high temperature-based BioC900/PA66 biocomposites were found to be higher than the low temperature-based BioC500/PA66 biocomposites (Table 1). The density of the biocarbon was then calculated by taking the total density of the formulation and the density of neat nylon 6,6, and then using simple algebra to determine the density of the two different biocarbons. The low-temperature biocarbon had a density of 1.438 g/cm^3^ and the high-temperature biocarbon had a density of 1.469 g/cm^3^. These densities are much lower than that of a traditional mineral filler such as talc which has a density of approximately 2.7 g/cm^3^.

**Figure 3:**
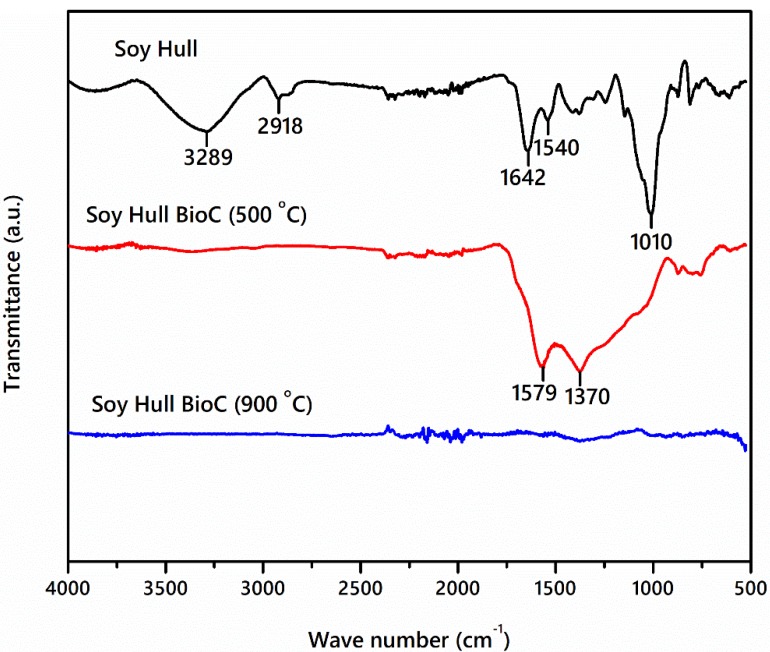
FTIR spectra of soy hull biomass, 500 °C soy hull biocarbon and 900 °C soy hull biocarbon. Wavenumbers of characteristic peaks are shown on the graph with units of cm^−1^.

Figure 3 presented the FTIR curves of the two pyrolyzed biocarbons. The peak at 1540 cm^−1^ corresponds to the aromatic –C=C– stretching due to the aromatic structure presence on the soy hull [41]. The sharp peak at 1010 cm^−1^ can be attributed to the ether –C-O-C– stretching bond [41]. The bands around 1650–1750 cm^−1^ can be due to the presence of free and esterified carboxyl groups [42]. It can be seen that the BioC500 preserved more functional groups from the raw soy hull biomass, (i.e., O-H bending at 1579 cm^−1^ and C-H bending at around 1370 cm^−1^ band) after pyrolysis. On the other hand, the BioC900 shows very limited functionality if any at all. These available functional groups could be possibly adhering to the PA66 end groups. The biocarbon could interact with nylon backbone through polar–polar interactions due to similar affinity towards each other [43]. The BioC900 should be more apt to bonding with a nonpolar molecule such as polypropylene due to the lack of functional groups as suggested by the study Behazin et al. [44]. It can also be assumed that decreasing pyrolysis temperature would yield even more functional groups akin to those on the raw soy hull but further testing is required to confirm this assumption. 

### 2.2. Fourier Transform Infrared Spectroscopy (FTIR)

**Figure 4:**
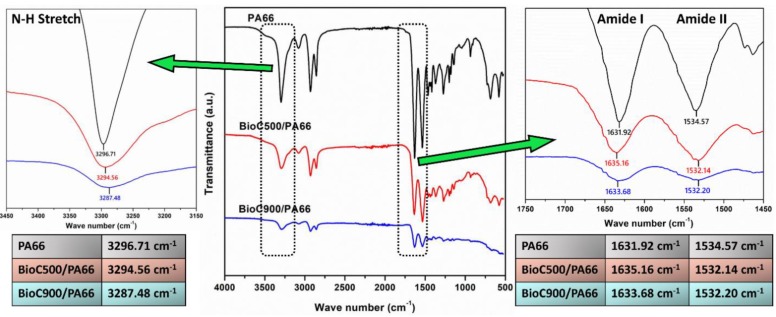
FTIR spectra of PA66 and its composites with low pyrolysis temperature and high pyrolysis temperature BioC.

The degree of interaction of the biocarbons with PA66 was further examined with FTIR (Figure 4). The details of the FTIR bands of PA66 functional groups have been well reported [45,46,47]. Our investigation only focuses on the main characteristics of PA66 i.e., N-H stretch at ~3300 cm^−1^, Amide I (C=O) peaks at 1631 cm^−1^ and Amide II (N-H) peak at ~1535 cm^−1^. It can be seen that the characteristic peaks of the PA66 was reduced after the incorporation of fillers. Greater peak intensity reduction was observed for high-temperature biocarbon incorporation. The N-H stretch band of the PA66 bond was observed to shift to a lower wavenumber (redshift). The BioC900 exhibited a higher shift (6 cm^−1^) than the BioC500 (2 cm^−1^). The shift of this band can be directly related to the hydrogen bonding formation between filler and PA66 [48]. This indicates that the high pyrolysis temperature biocarbon is forming stronger hydrogen bonds with PA66. There is a greater shift for the BioC500/PA66 as compared to BioC900/PA66 at the amide I band. A similar extent of shifting was observed at the amide II peak for both biocomposites. A small peak at the ~1537 cm^−1^ band from the soy hull biocarbon appears beside the amide II peak for both biocomposites which confirms the attachment of the BioC with PA66. 

### 2.3. Thermal Analysis and Characteristics

**Figure 5:**
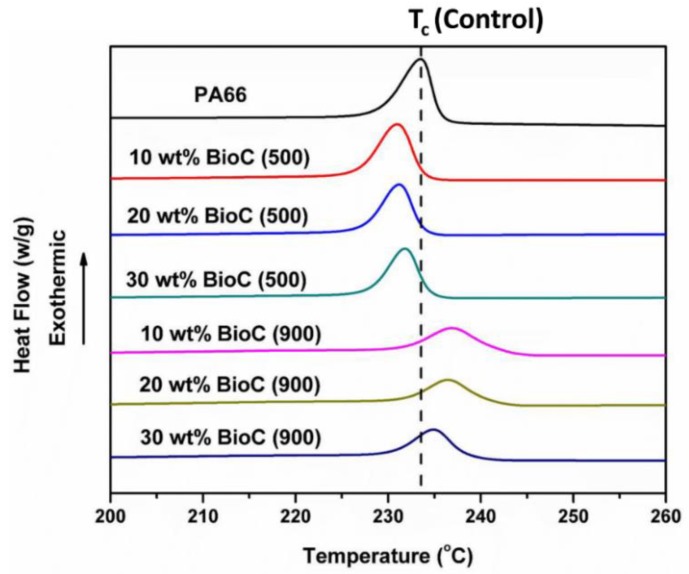
DSC cooling cycle of BioC/PA66 biocomposites showing crystallization temperature peaks.

Differential scanning calorimetry (DSC) measures the heat flow through a material and can be used to determine melting temperature (*T_m_*), crystallization temperature (*T_c_*), and also percent crystallinity (*X_c_*). Table 2 presents all the DSC data extracted from the DSC curves of PA66 and its composites and Figure 5 displays the influence of different pyrolysis conditions of biocarbon on the cooling curves of PA66. For the melting point, a slight reduction in melting temperature was observed after the incorporation of biocarbon in PA66. It decreased as the amount of biocarbon increased. Greater reduction in *T_m_* was observed with the higher pyrolysis temperature BioC900 as compared to lower pyrolysis temperature BioC500 in the second heating cycle. The reduction in *T_m_* to a lower temperature after the incorporation of fillers was due to the reduction in the crystallite size of the PA66 [49,50]. 

Both types of biocarbon affect the crystallization temperature in different ways. The *T_c_* peak decreased with the addition of BioC500 but increased with the addition of BioC900 at 10 wt% filler content. The *T_c_* of the biocomposite was shifted to a lower temperature with the addition of 10 wt% of BioC500 (Figure 5). On the other hand, the *T_c_* peak shifted to a higher temperature value with the addition of 10 wt% BioC900, with its value being 4 °C above that of the neat PA66. The *T_c_* of the biocomposites shifts towards that of neat PA66 with increasing filler content for both types of fillers, which indicates exceeding the optimum saturation point. The lowering of *T_c_* can be due to the disruption of the growth of perfect crystallites and chain arrangement during cooling as a result of strong chain mobility restrictions [51,52]. The presence of the BioC500 hinders the growth of α-crystals in PA66, while the *T_c_* shifting towards a higher temperature indicates the domination of heterogeneous nucleation as a result of the acceleration of chain arrangement with the presence of filler. The formation of imperfect crystals is evident in the subsequent heating process of the DSC curve (*T_m_* in second heating), where the *T_m_* of the composites are lower than the neat PA66 due to the early melting of the small and imperfect crystal structures of PA66. This indicates the BioC500 exhibited higher molecular interaction with PA66 as compared to BioC900 which aligns with the FTIR results.

The degree of crystallinity, *X_c_* is a measure of the amount of material that is in a crystalline structure rather than being amorphous. The formulation with the highest *X_c_* is 30 wt% BioC500 in the first heating curve, and the formulation with the highest crystallinity in the second heating curve is 30 wt% BioC900. The 30 wt% BioC900 also results in the highest average of crystallinity out of all 6 composite formulations. This is corroborated with the reduction in the *T_m_* of the biocomposites. While the presence of high pyrolysis temperature BioC900 in PA66 was found to favor the crystal growth process as indicated by the shift of the *T_c_* to a higher temperature. The BioC900 induces the heterogeneous crystallization growth in the PA66 as seen by the increase of *X_c_* in the second heating curve of DSC. 

**Figure 6:**
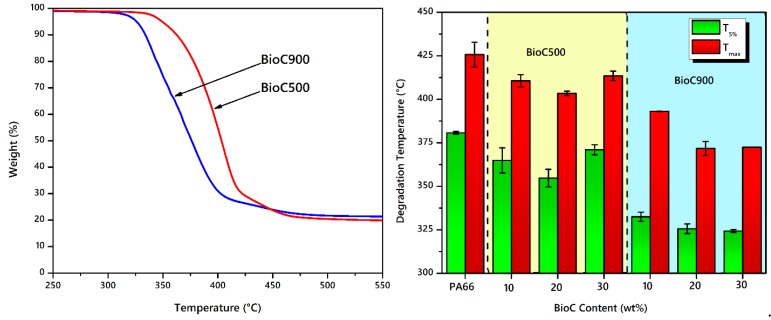
(**a**) Thermogravimetric analysis (TGA) curves of BioC/PA66 biocomposites at 20 wt% and (**b**) comparison of degradation temperature at 5% weight loss and maximum decomposition of BioC/PA66 biocomposites.

The TGA curves of both BioC500 and BioC900 reinforced PA66 biocomposites are presented in Figure 6a. It can be seen that the incorporation of BioC500 results in a higher thermal stability than BioC900. In comparison to the degradation temperature at 5% and maximum decomposition, the BioC500/PA66 exhibited higher degradation temperature by around 30 °C to 40 °C (Figure 6b). The same trend is observed in the T_5%_ values where BioC500/PA66 composites consistently have higher degradation temperatures than BioC900/PA66 composites. This difference in thermal degradation temperature is caused by the difference in interaction and structure formed between the biocarbons and the polymer matrix [53]. 

### 2.4. Dynamic Mechanical Analysis (DMA) 

**Figure 7:**
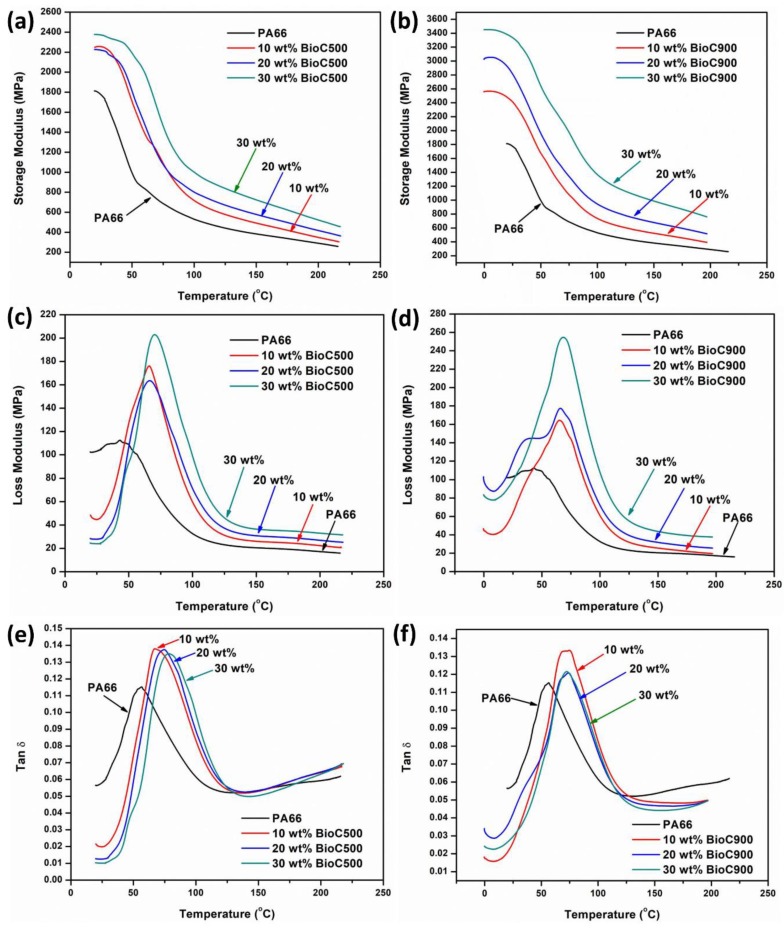
DMA curves of PA66 and its biocomposites with low and high pyrolysis temperature BioC at different weight contents (**a**) storage modulus, (**c**) loss modulus, (**e**) Tan δ of BioC500/PA66, and (**b**) storage modulus, (**d**) loss modulus, and (**f**) Tan δ of BioC900/PA66.

Figure 7 presented the effect of low and high pyrolysis temperature biocarbon on the dynamic storage modulus, loss modulus, and Tan delta (δ) of PA66. It can be seen that both types of biocarbons enhance the storage modulus of PA66 with increasing filler content throughout all temperature ranges (Figure 7a,b). However, the high-temperature biocarbon exhibited a higher storage modulus as compared to the low-temperature biocarbon. The effect is more vivid with increasing temperature. For instance, for the same filler loading of 30 wt% at 150 °C, the storage modulus of BioC900/PA66 is around 1000 MPa while the BioC500/PA66 was around 800 MPa. The higher storage modulus indicates a higher mechanical rigidity of the BioC900 as compared to BioC500. This can be due to the presence of higher amounts of higher packing carbon structure in BioC900 as compared to BioC500 as evidenced in the Raman spectrum. The loss modulus peak of the PA66 also appears to be more intense and sharpens with increasing content of both types of biocarbon (Figure 7c,d). This is due to the chain mobility restriction with increased filler content in the PA66 composites during deformation. The energy required to dissipate the heat and internal friction becomes larger due to the particle interruption between the PA66 chains. 

The chain movement restrictions of PA66 were further indicated in the shifting of the glass transition temperature, *T_g_* towards a higher temperature after incorporation of both types of biocarbon. The peak of Tan δ curves shows the α relaxation peak of PA66, which reveals the chain movement in the amorphous segment of PA66. For the BioC500, the *T_g_* increases relatively with increasing BioC content from 10 to 30 wt% (from 56 °C for neat PA66 to 67 °C, 74 °C, and 78 °C for 10, 20, and 30 wt% BioC respectively) (Figure 7e). There is no systematic enhancement in *T_g_* observed for the BioC900/PA66 biocomposites with increasing filler content (approximately 72 °C to 73 °C for the three 10, 20, and 30 wt% filler loading) (Figure 7f). This shows that BioC500 exhibited stronger interaction with PA66 which results in a higher shifting of *T_g_* as compared to BioC900. The relaxation of PA66 chains was interrupted by the presence of the biocarbon filler. Therefore, the PA66 demonstrated more elastic behavior than viscous with less damping ability after the addition of fillers. The restriction on the mobility of PA66 chains due to the interfacial bonding with the filler was also displayed in the increasing Tan δ peak value of the PA66 composite as compared to neat PA66 [49]. This observation also reported by Nagarajan et al. in a system of polytrimethylene terephthalate (PTT) with different size fractions of biocarbon which can be attributed to the higher viscoelastic energy dissipation of the biocomposites [54].

**Figure 8:**
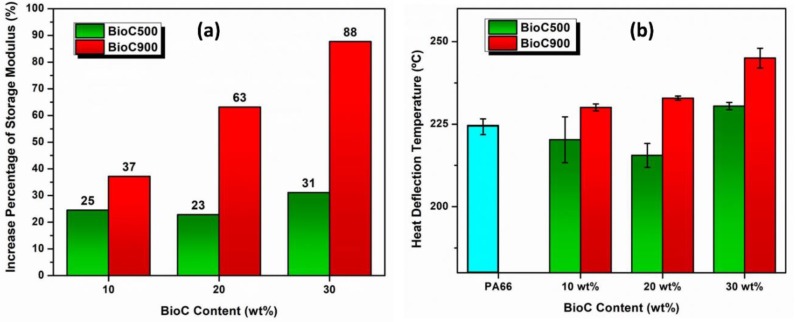
(**a**) Percentage increase in storage modulus over neat PA66. (**b**) Heat deflection temperature of BioC500/PA66 and BioC900/PA66 biocomposites.

Figure 8 presents the extraction of storage modulus at 20 °C of DMA test and the heat deflection temperature (HDT) of BioC/PA66 biocomposites as compared to neat PA66. It was observed that the storage modulus increased with all biocarbon formulations as compared to neat PA66. The 30 wt% biocarbon showed the highest storage modulus as compared to other content for both PA66 biocomposites. The largest increase in modulus was a result of the 30 wt% of BioC900/PA66 composite which yielded an increase of 88% over neat PA66 (Figure 8a). This is likely due to the increase in stiffness and rigidity of the biocomposites with the presence of biocarbon. The percentage increase in the storage modulus of PA66 increases with increasing biocarbon content (Figure 8a). The BioC500 showed much less increase in modulus due to less rigidity and stiffness as compared to BioC900. As discussed in the Raman spectroscopy results, the BioC900 possesses higher graphitized carbon content than disordered carbon content. A lower temperature biocarbon will have less stiffness and rigidity than biocarbon pyrolyzed at high temperatures due to the dominance of less ordered carbon. Therefore, the high-temperature biocarbon tends to have greater stiffness and rigidity which will result in these characteristics manifesting in the polymer composite. 

This is further supported by the mechanical properties of the biocomposites as shown in Table S1. The flexural strength of the PA66 was increased with the incorporation of the biocarbons. The BioC500/PA66 exhibited higher flexural strength and notched impact strength than the BioC900/PA66 due to the better adhesion. On the other hand, the flexural modulus of the BioC900/PA66 is higher than the Bio500/PA66 due to higher rigidity. Hence, the incorporation of low-temperature biocarbon in PA66 yielded higher flexibility, while high-temperature biocarbon displayed higher rigidity in the PA66-based biocomposites. 

As seen in Figure 8b, the HDT of PA66 increased with the addition of biocarbon in a manner mirroring that of the storage modulus. It was observed that the 30 wt% BioC900 had the highest HDT out of all the formulations. The BioC900 formulations had both higher storage moduli and HDT than the BioC500 formulations at the same filler content. These changes can be attributed to the different properties of the biocarbon in each formulation, where BioC500 has less graphitic carbon content than BioC900. 

### 2.5. Rheological Behavior 

**Figure 9:**
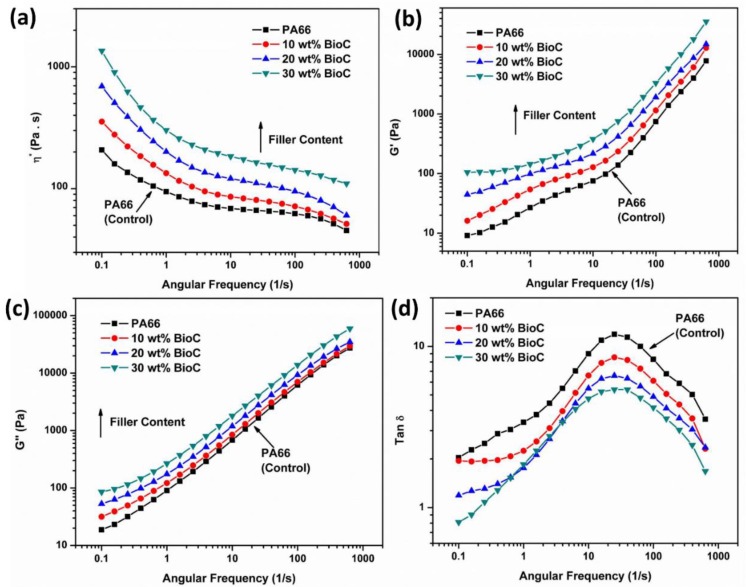
Rheological behavior of PA66 with different contents of 500 °C pyrolytic temperature of BioC. (**a**) complex viscosity; η*, (**b**) storage modulus; G′, (**c**) loss modulus; G″, and (**d**) Tan delta.

In order to examine the filler–matrix interaction, a frequency sweep test was carried out for the developed PA66 biocomposites with low and high pyrolysis temperature biocarbons. Unlike the Newtonian flow behavior of PA6 where the melt flow behavior is less reliant on the shear rate, the flow behavior of PA66 is much more sensitive to shearing action. The neat PA66 shows shear thinning behavior with decreasing complex viscosity when increasing the shear rate. The effects of low-temperature biocarbon, BioC500 on the rheological behavior of PA66 are shown in Figure 9. It was observed that the complex viscosity (η*), storage modulus (*G′*) and loss modulus (*G″*) of PA66 were enhanced with increasing biocarbon content from 10 wt% to 30 wt% (Figure 9a–c). The enhancement in complex viscosity of PA66 was found to be more substantial at low angular frequency. This enhanced viscosity of PA66 with BioC500 reflects good filler–matrix interaction in the composites system. These results are corroborated with the findings reported by Abdelwahab et al. and Ogunsona et al., where the complex viscosity of the PA6 was increased with the incorporation of carbonized miscanthus fiber [31,43]. Good filler–matrix network formation was observed from high shear rates to low shear rates in BioC/PA66 with the complete enhancement of the complex viscosity curves across the entire range. Regarding the damping factor, it was observed that the Tan δ of PA66 and its biocomposites exhibited peak value at around 50 rad/s angular frequency (Figure 9d). The Tan δ curve of the PA66 decreased with increasing biocarbon content. This indicates that the damping ability was reduced, where the elastic behavior is more pronounced than the viscous behavior after the incorporation of fillers. The energy loss is decreased with the presence of fillers due to superior mechanical restraints as a result of good filler–matrix interaction [55]. Therefore, the Tan δ (ratios of *G′* to *G″*) decreases with increasing biocarbon content. 

**Figure 10:**
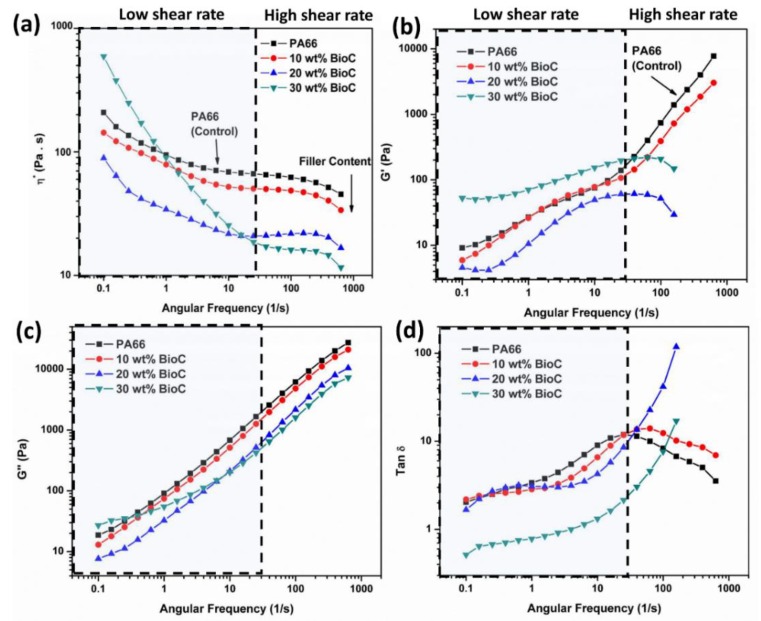
Rheological behavior of PA66 with different contents of 900 °C pyrolytic temperature of BioC. (**a**) Complex viscosity; η*, (**b**) storage modulus; G′, (**c**) loss modulus; G″, and (**d**) Tan delta.

Compared to the rheological behavior of BioC500 in PA66 biocomposites, the opposite trend in BioC900/PA66 was observed. The η*, *G′* and *G″* of PA66 were reduced after incorporation of high-temperature biocarbon, BioC900, except 30 wt% at low shear rates (Figure 10a–c). The trend was reversed at 30 wt% biocarbon where a higher increment in complex viscosity was observed at low angular frequencies as compared to high angular frequency (Figure 10a). The reduction in rheological behavior indicates that there is poor and limited filler–matrix interaction in the composite system. The better interaction of BioC500 over BioC900 with PA66 corroborates with the FTIR results from earlier which is due to the higher functionality presence on the BioC500 that could readily connect with the PA66 end groups at high temperature. This aligns well with our previously published work on PA6/biocarbon-based biocomposites where the incorporation of lower temperature biocarbon exerts a greater restriction on the flow mobility of the nylon chains due to stronger interactions with nylon as compared to high-temperature biocarbon [43]. 

The BioC900 acts as a lubricant in the PA66 matrix under high shear rate conditions which causes easy slippage between chains. As a result, the η*, *G*′, and *G*″ reduces with increasing biocarbon content. On the other hand, the complex viscosity was found to increase drastically under low shear frequency at 30 wt% biocarbon loading. The *G*′ and *G*″ curves of 30 wt% filler loading also deviate from typical curves and move towards plateau at low shear rate regions. The *G*′ becomes less sensitive with increasing frequency which shows solid-like behavior [56]. This indicates the presence of filler aggregation at high filler loading in the composite. The rheological behavior of the highly filled polymers is not following typical shear rheology curves due to the dominance of particle–particle contact and aggregation. Furthermore, the Tan δ curve of 30 wt% BioC/PA66 is low in value and moving towards less than 1.0 value which indicates severe filler agglomeration in highly filled biocomposites [56] (Figure 10d). By comparing both biocarbons under highly filled conditions, the BioC900 is showing higher particle agglomeration where it loses the filler–matrix network as shown in the drastic reduction in the storage modulus at high frequency shear rates (Figure 10b). The BioC500 is more interactive with the PA66 due to higher surface functional groups, which prevent and limit the occurrence of particle–particle aggregation which is dominant in the case of high-temperature BioC/PA66 biocomposite. The different rheological behaviors of both types of filler in PA66 can be due to their interaction with the PA66 structure in different manners as presented in FTIR analysis previously. The BioC900 shows a higher degree of interaction with N-H while the BioC500 tends to interact with amide I linkage. 

The rheology results demonstrate that both types of biocarbon fillers (high and low pyrolysis temperature) affect the PA66 behavior in different ways during melt processing. Depending on the situation and applications, the high-temperature biocarbon is more beneficial as a processing aid in injection molding and fiber spinning due to its lubricating behavior in the composites.

**Figure 11:**
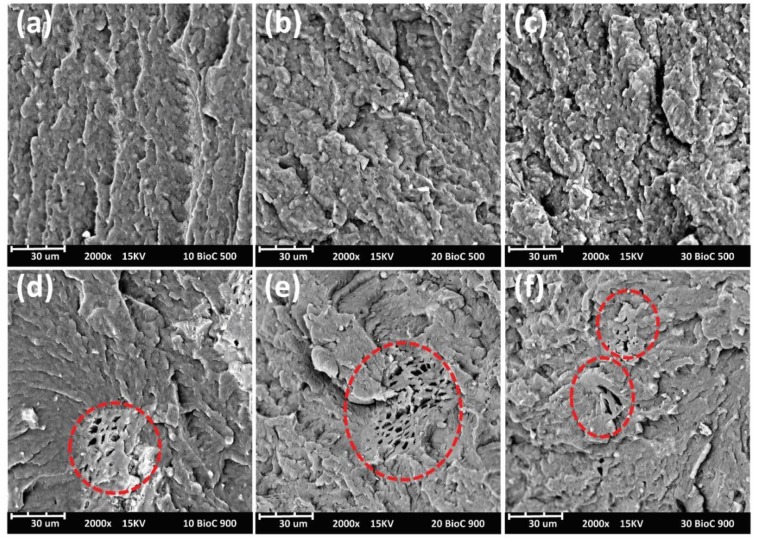
SEM images of the cryo-fractured morphology of BioC/PA66 composites with different biocarbon content. (**a**) 10 wt%, (**b**) 20 wt%, and (**c**) 30 wt% of BioC500/PA66, and (**d**) 10 wt%, (**e**) 20 wt%, and (**f**) 30 wt% of BioC900/PA66 biocomposites.

The cryo-fractured interfaces of both PA66 biocomposites are presented in Figure 11, it can be clearly seen that the BioC900/PA66 contains larger particles of biocarbon as compared to BioC500/PA66, which corroborates well with the particle size analysis. Overall, the PA66 reinforced with BioC500 shows uniform dispersion with less distinguishable phases between the filler and the matrix (Figure 11a–c). This phenomenon aligns with the trend in complex viscosity where the melt viscosity increases consistently with BioC500 filler content due to proper adhesion with PA66. 

## 3. Experimental Details 

### 3.1. Materials

Nylon 6,6 (Zytel 101L NC010, PolyOne, Mississauga, Canada), an unreinforced, injection grade nylon (density ~1.14 g/cm^3^) was used in this study. Soy hulls were provided by Nieuwland Feed & Supply Ltd from Elora, Ontario, Canada, they are in the form of flakes, approximately 3 mm in diameter. The soy hull biomass contains approximately 42% cellulose, 18% hemicellulose and 2% lignin and the complete characterization procedure of this biomass can be found in our previous paper [16]. 

### 3.2. Biocarbon Preparation

**Scheme 1:**
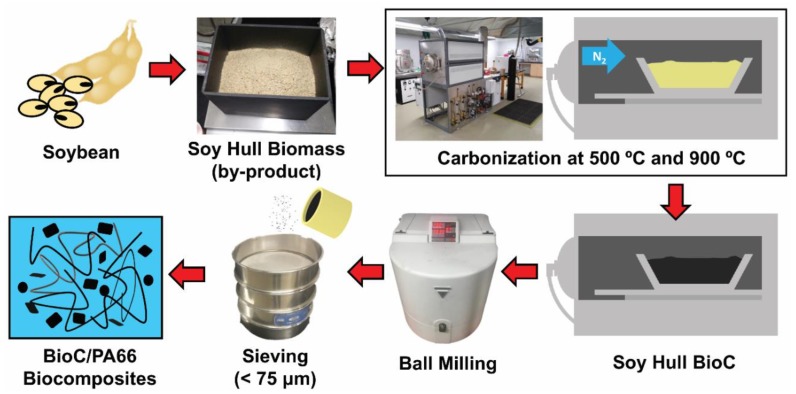
The preparation procedure of biocarbon as filler in PA66.

Soy hulls were dried for three days before pyrolysis to ensure low moisture content. Pyrolysis was carried out in a Carbolite GLO 10/11-1G annealing furnace. Soy hulls were pyrolyzed at 500 °C and 900 °C with a dwell time of one hour, under a nitrogen purge rate of 100 LN/h. The carbonized soy hull yield was approximately 29% from the 500 °C pyrolysis and 25% from the 900 °C pyrolysis which is close to the reported values found by Quosai et al. [16]. In order to reduce the size and ensure the consistency, the pyrolyzed soy hulls were then ball milled in a Fritsch Pulverisette 5 with 100 zirconium oxide balls (1 cm diameter) in 500 mL zirconium oxide jars for 2 h in 30 min intervals with reversing direction. The biocarbon was then sieved in a Ro-Tap Sieve Shaker. Only the particle sizes of <75 µm were collected and used for both 500 °C and 900 °C biocarbons as filler in PA66. The complete biocarbon preparation process is presented in Scheme 1. The obtained biocarbon is hereafter referred to as BioC500 and BioC900 as according to their pyrolysis temperature. 

### 3.3. Biocomposites Preparation

PA66 pellets were dried at 80 °C in an oven overnight before processing. The neat PA66 and its biocomposites (with 10, 20, and 30 wt% for both 500 °C and 900 °C soy hull BioC) were produced using a 15 cc DSM Micro-compounder with a processing temperature of 280 °C. The melt mixing was left for 2 min and the molten mixture was then injected into the mold with a 30 °C mold temperature and a pressure of 10 bar. The physical appearance of the developed PA66 biocomposite samples with both 500 °C and 900 °C soy hull BioC are presented in Figure S1. All samples were kept in a sealed zip lock bag for 48 h and in conditions according to ASTM D618 prior to processing. 

### 3.4. Characterization 

Fourier transform infrared spectroscopy was done on a Nicolet 6700 FTIR Spectrometer with 64 scans and a resolution of 4 cm^−1^ in order to see the functional groups of both the high and low temperature pyrolyzed biocarbon and the composite’s adhesion. The relative amount of disordered carbon to the graphitized carbon of the carbonized soy hull at different pyrolysis temperature was confirmed using Raman spectroscopy from Thermo Fisher Scientific. The laser power was set at 5 mW at a wavelength of 532 nm and the aperture was set to a 50 µm pinhole. Density measurements were carried out on an Alfa Mirage Electronic Densimeter in order to compare densities of the various formulations. 

Differential scanning calorimetry was used to determine the various thermal transitions of the samples and was done on a DSC Q200 from TA Instruments. Samples were held isothermally at 30 °C for 2 min, then heated at a rate of 10 °C/min to 290 °C, held for 3 min, then cooled at a rate of 10 °C/min back to 30 °C, the sample was then heated back to 290 °C at the same 10 °C/min rate and once again held isothermally for 3 min. 

TGA was done using a TGA Q500 by TA instruments. Approximately 15 mg of material was heated up to 700 °C at a rate of 10 °C/min while the chamber was purged with nitrogen at a rate of 60 mL/min. The mass of the sample was recorded as the temperature increased in order to find the thermal degradation properties of the materials. 

Heat deflection temperature was measured using a DMA Q800 by TA Instruments. The sample dimension of 50 mm × 12 mm × 3 mm (*l* × *w* × *t*) was set up in 3-point bending clamp mode in the chamber. The stress of 0.455 MPa was used to evaluate the HDT of the samples. The target temperature was 240 °C with a heating rate of 2 °C/min and the machine set to abort heating if the sample deflection reached 250 µm according to ASTM D648. 

Dynamic mechanical analysis was also completed on the DMA Q800. The sample dimensions of 50 mm × 12 mm × 3 mm (*l* × *w* × *t*) were used in dual cantilever clamp mode at a frequency of 1 Hz. All the samples were heated from 20 °C to 220 °C with a heating rate of 3 °C/min. 

The rheological behavior of the PA66 and its biocomposites were measured using an Anton Paar rheometer model MCR-502. The test samples were cut into a disk shape with a diameter of 25 mm and placed between the parallel plate setup with a 1 mm gap. The rheology test was performed within a 1% strain with a frequency sweep range of 628 to 0.1 rad/s from high to low shear frequency. To avoid sample degradation, the test was carried out at 280 °C (processing temperature of nylon 66) under a nitrogen atmosphere with a constant flow of nitrogen in the test chamber. The shear rheology properties (i.e., complex viscosity, storage modulus, loss modulus and damping factor) were acquired and reported accordingly.

Flexural testing was performed on a Universal Testing Machine (Instron 3382) with a crosshead speed of 14 mm/min, and a span length of 52 mm in accordance with ASTM D790. Notched impact testing was carried out on a Zwick/Roell Hit25P Plus pendulum impact tester following the ASTM D256 test (method A) using a 2.75 J hammer.

The interfaces of the PA66-based composites were examined with a Phenom ProX desktop scanning electron microscope from Phenom World, Netherlands. The samples were immersed in liquid nitrogen and bent to break when brittle. All the cryo-fractured surfaces of the samples were coated with a thin gold layer using a Cressington Sputter Coater 108 auto vacuum sputter chamber prior to imaging.

## 4. Conclusions

PA66-based biocomposites with low-cost carbonaceous soy hull natural fibers (co-product from soybean industry) were successfully prepared through twin-screw extrusion and injection molding. The BioC500 preserved a higher number of functional groups as compared to BioC900 after pyrolysis. The ratio of I_D_/I_G_ of BioC900 is less than BioC500 which indicates that higher graphitic carbon content can be obtained with higher temperature pyrolysis. The specific gravity of BioC900 was also higher than the BioC500 due to a higher amount of orderly packed carbon. Fourier transform infrared spectroscopy (FTIR) results indicated that both biocarbons interact with PA66 backbone through hydrogen bonding in different ways. BioC900 showed higher reactivity with N-H stretching, while BioC500 exhibited higher reactivity with amide I (C=O stretching) linkage. 

The BioC500 interrupts the crystallite growth of PA66 due to strong bond connection while the BioC900 promotes heterogeneous crystallization in PA66. The BioC500/PA66 exhibited a higher decomposition temperature and thermal stability than the BioC900/PA66 biocomposites due to the difference in their intrinsic structures and interactions with the PA66. Dynamic mechanical analysis showed an increase in glass transition temperature of more than 10 °C in the BioC/PA66 biocomposites over PA66 due to molecular chain restriction after incorporation of biocarbon. The rheology results demonstrate that BioC500 is more interactive with the PA66 due to higher surface functional groups which prevent and limit the occurrence of particle–particle aggregation that are dominant in the case of BioC900/PA66 composites. The melt viscosity increases with the incorporation of BioC500, while the reverse trend was observed for BioC900. The high pyrolysis temperature soy hull biocarbon is more beneficial as a processing aid in injection molding and fiber spinning due to its lubricating behavior during melt compounding. Both storage modulus and heat deflection temperature (HDT) of PA66 increase with increasing biocarbon content. The incorporation of BioC900 results in a significantly higher increase in the storage modulus and HDT than the incorporation of BioC500 in PA66 biocomposites.

The good interaction of biocarbon and PA66 enable the feasible use of carbonized natural fibers as an alternative to other cost-reducing fillers in PA66 for automotive parts, such as talc and calcium carbonate. Biocarbon has the advantage of being sustainable and low in cost and density compared to other materials that are commonly used in the market today. This work demonstrated a possible low-cost PA66-based biocomposites with the exploitation of an agricultural co-product which progresses towards better and sustainable development.

## Figures and Tables

**Table 1:** Measured density of PA66 and its biocarbon-based composites at different filler content.

Samples	Density (g/cm^3^)
PA 6,6	1.137 ± 0.002
10 wt% 500 BioC	1.162 ± 0.012
20 wt% 500 BioC	1.194 ± 0.003
30 wt% 500 BioC	1.227 ± 0.001
10 wt% 900 BioC	1.173 ± 0.003
20 wt% 900 BioC	1.206 ± 0.002
30 wt% 900 BioC	1.232 ± 0.004

**Table 2:** DSC data of PA66 and its composites with standard deviation in parenthesis, Heat 1 includes the first heating and cooling curve.

First Heating					Second Heating		
Sample	T_m_ (°C)	∆H_m_ (J g^−1^)	X_c_ (%)	T_c_ (°C)	T_m_ (°C)	∆H_m_ (J g^−1^)	X_c_ (%)
PA 6,6	264.03 (0.82)	76.91 (2.39)	30.11 (0.94)	233.04 (0.63)	262.62 (0.04)	74.27 (0.27)	29.08 (0.11)
10 wt% BioC (500)	262.76 (0.03)	65.23 (1.98)	28.38 (0.86)	230.97 (0.01)	261.79 (0.39)	65.91 (0.78)	28.67 (0.34)
20 wt% BioC (500)	262.67 (0.17)	58.21 (0.72)	28.49 (0.35)	231.25 (0.54)	261.33 (0.17)	60.01 (0.10)	29.37 (0.05)
30 wt% BioC (500)	262.49 (0.95)	51.76 (0.26)	28.95 (0.15)	231.94 (0.13)	260.93 (0.10)	51.43 (0.20)	28.77 (0.11)
10 wt% BioC (900)	262.34 (0.25)	63.51 (2.35)	27.63 (1.02)	237.01 (0.17)	260.96 (0.25)	68.35 (0.51)	29.73 (0.22)
20 wt% BioC (900)	261.91 (0.57)	55.08 (1.49)	26.95 (0.73)	236.52 (0.35)	259.50 (0.18)	59.84 (0.03)	29.29 (0.01)
30 wt% BioC (900)	262.96 (0.29)	50.22 (1.66)	28.09 (0.93)	235.02 (0.06)	258.02 (0.14)	54.44 (0.92)	30.45 (0.51)

## Supplementary Materials

The following are available online at https://www.mdpi.com/1420-3049/25/6/1455/s1, Figure S1: Digital photo of the prepared biocomposite samples (a) Neat PA66, (b) 20 wt% BioC500 reinforced PA66 and (c) 20 wt% BioC900 reinforced PA66. Table S1: Mechanical Properties of PA66/BioC composites at 20 wt%. Video S1: title
