# Supplementary material for: Underutilized Agricultural Co-Product as a Sustainable Biofiller for Polyamide 6,6: Effect of Carbonization Temperature
Source: Molecules. 2020 Mar 24;25(6):1455. doi: 10.3390/molecules25061455 (PMC7146422; doi:10.3390/molecules25061455)
Supplement: Supplementary file 1 [file molecules-25-01455-s001.zip › Supplementary Information molecules-732562.docx]

Underutilized agricultural co-product as a sustainable biofiller for polyamide 6,6: Effect of carbonization temperature

Thomas Balint ^1^, Boon Peng Chang ^1^, Amar K. Mohanty ^1,2,^* and Manjusri Misra ^1,2,^*

^1^ Bioproducts Discovery and Development Centre, Department of Plant Agriculture, Crop Science Building, University of Guelph, 50 Stone Road East, Guelph, Ontario N1G 2W1, Canada.

^2^ School of Engineering, Thornbrough Building, University of Guelph, 50 Stone Road East, Guelph, Ontario N1G 2W1, Canada.

*Corresponding authors email: [mohanty@uoguelph.ca](mailto:mohanty@uoguelph.ca) , [mmisra@uoguelph.ca](mailto:mmisra@uoguelph.ca)

**Supplementary Information**

**Table S1.** Mechanical Properties of PA66/BioC composites at 20 wt%

| Sample | Flexural Strength (MPa) | Flexural Modulus (GPa) | Notched Impact Strength (J/m) |
| --- | --- | --- | --- |
| Neat PA66 | 121.95 ± 1.14 | 3.126 ± 0.038 | 39.76 ± 2.00 |
| BioC500/PA66 | 135.82 ± 0.82 | 3.528 ± 0.037 | 33.47 ± 2.13 |
| BioC900/PA66 | 133.22 ± 1.28 | 4.075 ± 0.101 | 22.93 ± 0.96 |


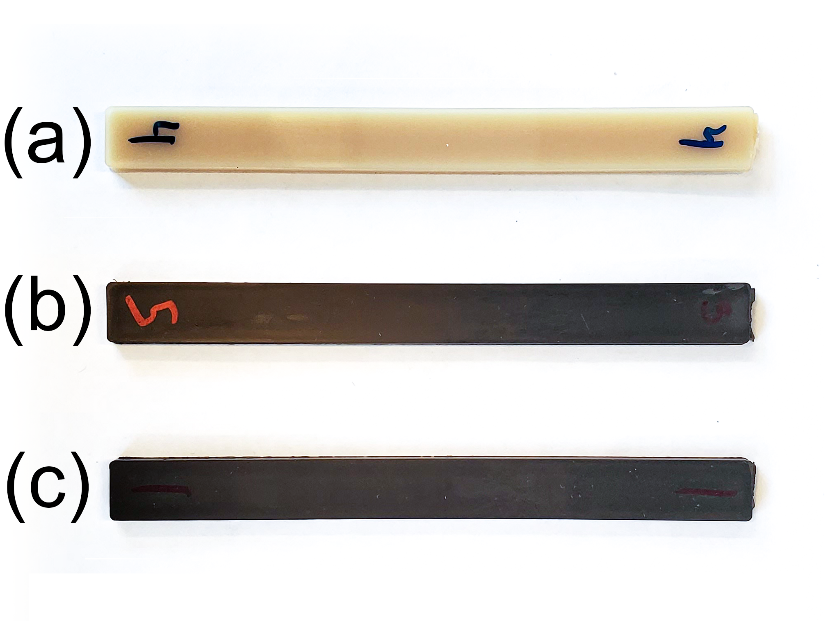


**Figure S1.** Digital photo of the prepared biocomposite samples (a) Neat PA66, (b) 20 wt% BioC500 reinforced PA66 and (c) 20 wt% BioC900 reinforced PA66.
