# Supplementary figures and images for: Underutilized Agricultural Co-Product as a Sustainable Biofiller for Polyamide 6,6: Effect of Carbonization Temperature
Source: Molecules. 2020 Mar 24;25(6):1455. doi: 10.3390/molecules25061455 (PMC7146422; doi:10.3390/molecules25061455)

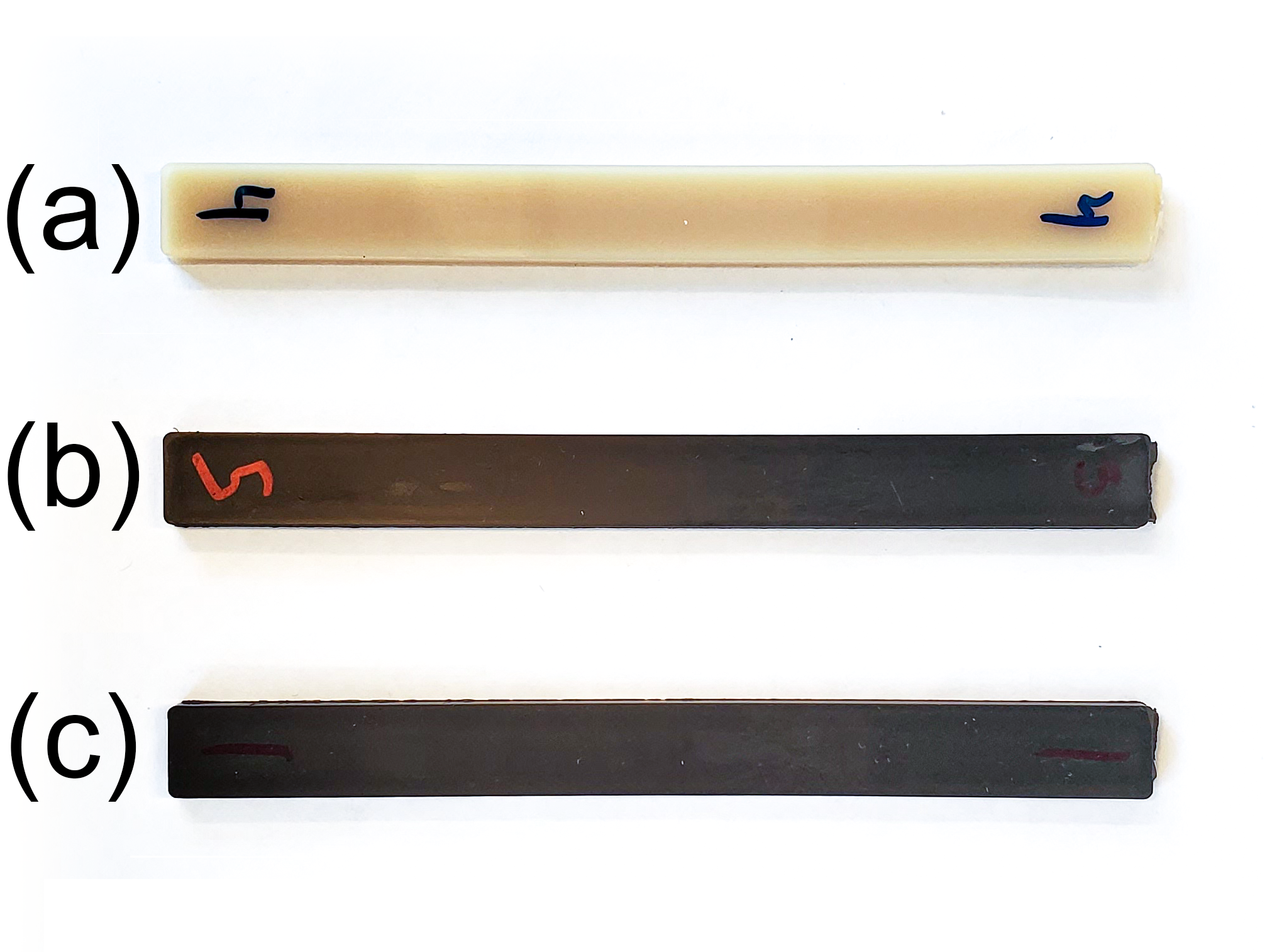

Supplement: Supplementary file 1 [file molecules-25-01455-s001.zip › Figure S1 Photo of Samples.png]
